# Process Parameters of High Frequency Welding

**DOI:** 10.3390/ma17020517

**Published:** 2024-01-21

**Authors:** Dubravko Rogale, Snježana Firšt Rogale, Željko Knezić, Siniša Fajt, Daniel Časar Veličan, Nikolina Jukl

**Affiliations:** 1 Department of Clothing Technology, University of Zagreb Faculty of Textile Technology, 10000 Zagreb, Croatia; dubravko.rogale@ttf.unizg.hr (D.R.); daniel.casar.velican@ttf.unizg.hr (D.Č.V.); nikolina.jukl@ttf.unizg.hr (N.J.); 2 Department of Textile Design and Management, University of Zagreb Faculty of Textile Technology, 10000 Zagreb, Croatia; 3 Department of Electroacoustic, University of Zagreb Faculty of Electrical Engineering and Computing, 10000 Zagreb, Croatia; sinisa.fajt@fer.hr

**Keywords:** high frequency welding, process parameters, anode current, welding time, coupling capacitor

## Abstract

High frequency (HF) welding of polymer materials is increasingly used in modern manufacturing processes. The literature on HF welding process parameters was reviewed and it was found that 3–5 basic welding parameters were considered, which is insufficient for the scientific study of HF welding of polymeric materials. This article presents the mathematical expressions for the evaluation combining 17 influential parameters. For the first time, the specific and latent heat of the welded polymer material were used. The breaking forces of welds made by RF welding are investigated by varying the anode current, the coupling capacitor, and the exposure time of the HF electromagnetic fields. It was found that the amount of HF energy supplied depends on the breaking forces of the weld. A characteristic inflection point was also observed in the graph of the dependence of the breaking forces on the percentage of the coupling capacitor and the anode current. During elongation, it was observed that the weld is separated by peeling before the inflection point and breaks after the inflection point by tearing at the extruded edges of the weld. If the HF energy is applied to the weld for too long, there will be excessive melting of the material in the weld, thinning of the weld, unfavourable appearance of the extruded edges and electrical breakdown, and a drastic drop in the breaking force.

## 1. Introduction

The use of high frequency (HF) electromagnetic fields for welding apparel is associated with high-tech welding techniques, such as ultrasonic welding, welding with hot wedges (conduction), hot air (convection), and welding with infrared lasers. These techniques are replacing the traditional techniques for joining apparel, namely sewing and gluing, due to their favourable properties such as lower energy consumption, high productivity, and environmental awareness. They are characterised by the exceptional strength of the welds produced as well as the air and water impermeability and are used for almost all joining techniques in clothing technology.

The oldest available research paper on high frequency heating dates back to 1944 and is entitled High frequency Heating. This paper describes not only the physical aspects of the heat phenomenon in treated materials, the technique for generating an HF electric field that causes rapid and uniform heating through the depth of the material, as well as the drying of wood laminates for the aircraft industry, the drying of agricultural raw materials and foodstuffs, diathermy (heat treatment of the human body at low wavelengths), but also the possibility of joining flat textile products [1]. Later, in 1946, a new paper entitled Radio Frequency Heating was published, covering the same topic as the previous one. This paper describes how HF vibration, induction, and electrical heating can be achieved [2]. The meaning of this article is that a real capacitor (represented by a polymeric material as the dielectric and machine electrodes as the capacitor plates) is represented as an equivalent scheme of a parallel circuit between an ideal capacitor and a real resistor where current is converted into heat. On the basis of such an equivalent scheme, in this paper the phase change between current and voltage in a dielectric is mentioned for the first time and the equation for the power dissipation in the material is used, which depends on the frequency, the tangent of the phase change angle, the square of the input voltage, and the dielectric properties of the treated material. In 1974, the DIN 16960 norm (DIN—Deutsches Institut für Normung) entitled Welding of Thermoplastics Principles defined practically all joining techniques for thermoplastics and laid down the methods and basic parameters of HF welding [3]. It states that the welding techniques are suitable for thermoplastic materials with a dielectric dissipation factor (DDF), also called loss angle (tgδ), equal to or higher than 0.01, measured at a frequency of 1 MHz and a temperature of 20 °C. The weld is heated between electrode plates, which form a capacitor, while the material is under a certain compressive force. The heat develops inside the material, the electrodes remain cold, and the maximum temperature develops in the middle of the welded seam of the materials when the layers of the materials to be joined have the same thickness. This technique is mainly used for welding foils and flat textile materials. The welding frequency of 27.120 MHz ± 0.6% is specified in the standard, the elements of welding techniques and welds are shown, but other process parameters are not mentioned. Apart from the two parameters mentioned, the standard contains a list of basic welding parameters: temperature, velocity, and pressure force, but the values of the parameters are not given.

Later books and guides were published that dealt with the subject of HF welding techniques. Dixon and Grewell mention some of the most important parameters for HF welding, namely the welding time (2–5 s), the material thickness (0.03–1.27 mm), and the chemical composition of thermoplastic materials [4]. They mention five main activities of welding (positioning of the specimen, loading pressure of the electrode, influence of the HF electromagnetic fields, cooling phase, and disposal procedure) and give a time interval for each activity, of which the HF electromagnetic fields’ influence on time (1–5 s) is the most important. They also give the mathematical expressions for the evaluation of the power dissipated in the dielectric as well as the power required to weld one square centimetre of the weld seam. It also mentions the main properties of the materials that can be welded using the HF technique, such as a high dielectric constant, dielectric losses and resistance to electrical arc, a list of materials with their weldability grades, and the most common defects that can occur during HF welding. Wu [5] described the technique of microwave welding, which is rarely available due to its high frequencies. Park and Grewell produced a detailed guide for the selection of HF welding techniques [6]. Pokharel and Karki [7] describe in great detail the dielectric properties of materials that are very important for HF welding. Troughton [8] describes a group of the most important welding parameters (output power of the machine, time of heating and cooling, thickness of the material, pressure and temperature of the upper electrode) and some basic properties but does not give the values of the parameters. Jones [9] encyclopaedically lists the strengths and weaknesses of HF welding and provides a table of suitable materials for HF welding divided into five categories. He describes and specifies the values most frequently used in practice for the welding time, the specific pressure of the electrode during welding, the voltage of the electrode, the frequency of the generator, and the temperature of the electrode as a guide for practical application and as a guide for the perception of the order of size of the parameters. He also specifies the area of the weld seam that can be achieved with a power of 1 kW. Pourmohammadi [10] deals with the problems of welding non-woven flat products with particular reference to HF welding technology, but he only mentions the frequency (27.12 MHz) and the thickness of the materials (<1.5 mm).

Shah [11] investigated the possibilities of using HF technology for welding samples used in medicine at a welding frequency of 27.12 MHz and gives a table of materials indicating the values of dielectric losses and the degrees of suitability of the welding materials which are divided into four classes. Bajsić et al. [12] have presented the changes in loss angles as a function of their dependence on the temperature change, which is very significant for the technique of HF welding when temperatures rise from room temperature to a dozen °C. Pierlot [13] gives the basic characteristics of HF welding and the processing parameters and only mentions that 10 to 30 square centimetres of material area can be joined with 1 kW HF power. Wu et al. [14] extended the research on the behaviour of the dependence of materials on electrical permittivity and loss angle in terms of frequency. Ingle and Deshmukh [15] published a table of thermoplastic materials with the abbreviations of the names, the welding temperatures, and the specific density of the materials. Hollande et al. [16] performed tests on the behaviour of dielectric properties when welding thermoplastic polyurethane at a frequency of 27.12 MHz. They mention formulae for calculating the complex permittivity and the cooling time, as well as their setup for determining the breaking forces of welds using the so-called T-peel test of tearing. With regard to the other process parameters, they state that the voltage developed at the electrode is 8 kV and the working pressure is 5 bar. The 3M guide [17] describes HF welding of reflective tapes on clothing with a frequency of 27.12 MHz, a material thickness 0.3 mm, and a welding area of 10 to 20 square centimetres at a power of 1 kW, a welding time of 1 to 6 s and a method for visual evaluation of the weld as well as ways of testing the strength with a representation of the factors that can cause delamination of the welds, damage to the welds if too much heat energy is injected into the weld or an electrical arc. Yousefpour et al. [18] describe the basic methods of so-called dielectric welding and mention the importance of the dielectric loss factor and the power of the HF generator, the material thickness, the welding area, the material properties, the welding time, and the pressure during welding. Grewell et al. [19] only give values for the material thickness of 0.03 to 1.27 mm, a frequency of 27.12 MHz, and the required relative dielectric constant of the material, which must be greater than 2. Other process parameters they specify are voltage, welding time, material properties, pressure force, and vertical displacement of the electrode. Mitelea et al. [20] deal with the optimization of welding parameters by changing the anode current of 2.5 to 5 mA, the welding time of 1 to 3 s, the welding pressure and cooling from 5 bar, and the cooling time from 2 s in three groups. The authors evaluated the optimum process parameters achieved by increasing the strength of the welds. Podržaj et al. [21] investigated changes in anode current and electrode displacement during welding as a function of welding time. They also provide a list of physical constants and process parameters, as well as mathematical expressions for the evaluation to calculate the power requirement per unit volume of the workpiece material and required voltage. Patil et al. [22] carried out an interesting work on the effect of frequency changes on the dielectric constant in the range of 1 to 500 MHz used in radio frequency and microwave heating of nanotubes in thermoplastic polymer materials. Amanat et al. [23] investigated the air and water permeability of welds made using HF electromagnetic fields in the field of hermetic encapsulation of medical devices and gave the properties of a large number of high-technology welding techniques. The paper focuses on the type of materials to be welded and the required strength of welds for hermetic encapsulation. Sano et al. [24] mention the use of HF welding technology in the aircraft and automotive industries. The special feature is the use of frequencies of 40 MHz to produce weld seams instead of the usual frequencies. They tested the dielectric constant of materials in the range of 300 MHz, which are generally constant, but the dielectric loss angle decreases with the applied frequency. Yang et al. [25] used the HF welding at a frequency of 27.12 MHz and investigated the welding temperature, welding time, welding pressure anode current and anode voltage, and tested the welding strength using the T-peel test. This is the only paper in which the authors point out the problem of the effects of electromagnetic fields on conventional and electronic measurement devices during the welding process. Čebular et al. [26] determine the quality of the weld seam when welding PVC films. They use the material thickness, five groups of the HF power used, and the anode current and then test the strength of the welds using the T-peel test. They concluded that the quality of the HF welding technique can be classified by measuring the thickness of the weld. Kaappa et al. [27] demonstrated a new application of HF welding technology in the integration of electronic components into flat textile products. They use the type of material, the specific pressure of the welding electrodes, the welding time and cooling as well as the percentage of the power of the HF generator. The results of the test elucidated the effect of the HF electromagnetic fields on the quality of the integrated electronic components in textiles. The properties of all HF welding techniques are being investigated. The process parameters used are anode current and anode voltage, pressure, HF power level, welding area, and material type and thickness [28].

Due to the lack of a complete description of all process parameters observed in the literature, the authors provide a complete mathematical expression for the evaluation of all dependencies of influencing parameters for the HF welding of polymer materials. The mathematical expressions for the evaluation are based on the systematic linking of all previously known parameters by mathematical equations using basic physical knowledge.

### Process Parameters of HF Welding and Their Interdependence

**Figure 1:**
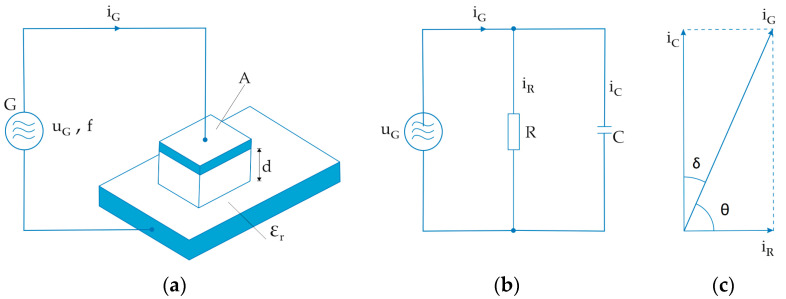
High frequency heating of polymer material: (**a**) principle of high frequency heating; (**b**) equivalent circuit; (**c**) phase graph of the currents of the equivalent circuit.

High frequency welding of polymer materials is carried out with the aid of an HF alternating electromagnetic field in which the material to be welded is located and in which heat is generated under the effect of the field. The material to be welded is positioned between the upper movable electrode and the lower plate, which is a fixed electrode. The electrodes are made of metal (aluminium or brass) and are generally a capacitor with an upper plate (upper moving electrode), a lower plate (fixed electrode, worktable), and a dielectric between the surfaces (material to be welded). Figure 1a shows the circuit of the capacitor with the upper plate of area A (upper movable electrode), the material to be welded with relative dielectric constant ε_r,_, and the lower plate (lower electrode or worktable WT). A high frequency alternating current generator G with a frequency f of normally 27.12 MHz is connected to the plates of the capacitor formed in this way (Figure 1a). The voltage u_G_ of the generator is relatively high, normally around 800 to 2000 V, at which a current i_G_ flows through the capacitor, which in practice reaches values between 0.2 and 0.6 A. The power of the HF generator used in clothing technology reaches values of up to 800 W for shorter weld seams on thinner materials (e.g., raincoats) and can be increased to values of 4 kW for longer and more durable weld seams on thicker materials (tarpaulins, sunshades).

Any real capacitor can be represented as an equivalent or substitute scheme circuit consisting of a parallel connection of the resistor R, through which the current i_R_ flows, and an ideal capacitor through which the current i_C_ flows (Figure 1b).

From a technological point of view, it is interesting and significant that the electrical power converted into heat during the exposure time of the high-frequency alternating field manifests itself as power dissipation at a resistor R, Figure 1b, and its value is the same:

(1) $$P=u_{G}\cdot i_{R}$$

where:

P—electrical power [W]
u_G_—generator voltage [V]
i_R_—current resistor [A]

Using the phase graph, the current flowing through the resistor that performs the work can be calculated using the equation:

(2) $$i_{R}=i_{G}\cdot \cos\theta$$

where:

i_R_—current resistor [A]
i_G_—current generator [A]
θ—phase angle of the displacement [rad]

So, the power of the alternating current is therefore calculated from the equation:

(3) $$P=u_{G}\cdot i_{G}\cdot \cos\theta$$

From Figure 1c, it can also be seen that:

(4) $$i_{C}=i_{G}\cdot \sin\theta \;\to {\;i}_{G}=\frac{i_{C}}{\sin\theta }$$

Substituting expression (4) into expression (3) gives:

(5) $$P=u_{G}\cdot \frac{i_{C}}{\sin\theta }\;\cdot \cos\theta \;\to \;P=u_{G}\cdot i_{C}\cdot \cot g\theta$$

From Figure 1c it can also be seen that:

(6) θ=90°−δ

Substituting expression (6) into expression (5) gives:

(7) $$P=u_{G}\cdot i_{C}\cdot \cot g\left(90{}^{\circ}-\delta \right)$$

Knowing that:

$$\cot g\left(90{}^{\circ}-\delta \right)=\mathrm{tg}\delta$$

Expression (7) can be written as:

(8) $$P=u_{G}\cdot i_{C}\cdot \mathrm{tg}\delta$$

Capacitive reactance of the capacitor X_c_ is equal to:

(9) $$X_{c}=\frac{1}{\omega \cdot C}=\frac{1}{2\cdot \pi \cdot f\cdot C}$$

where:

X_C_—capacitive reactance [Ω]
ω—angular frequency [rad]
C—capacity [F]
f—frequency of the HF generator [Hz]

The determination the current flowing through the capacitor can be written follows:

(10) $$i_{C}=\frac{u_{G}}{X_{c\;}}=\frac{u_{G}}{\frac{1}{2\cdot \pi \cdot f\cdot C}}=u_{G}\cdot 2\cdot \pi \cdot f\cdot C$$

Substituting expression (10) into expression (8) gives:

(11) $$P=u_{G}\cdot 2\cdot \pi \cdot f\cdot C\cdot u_{G}\cdot \mathrm{tg}\delta =u_{G}^{2}\cdot 2\cdot \pi \cdot f\cdot C\cdot \mathrm{tg}\delta \;$$

During the HF welding the upper electrode of area A, which welds the polymer material of thickness d and relative dielectric constant ε_r_, together with the lower electrode forms the capacitor with the capacity:

(12) $$C=\varepsilon_{0}\cdot \varepsilon_{r}\cdot \frac{A}{d}\;$$

Substituting expression (12) into expression (11), the expression for calculating the power level P from the voltage, i.e., the voltage of the upper electrode is obtained:

(13) $$P=u_{G}^{2}\cdot 2\cdot \pi \cdot f\cdot \varepsilon_{0}\cdot \varepsilon_{r}\cdot \frac{A}{d}\cdot \mathrm{tg}\delta \;$$

In practice, it is better to use an expression based on the generator current rather than generator voltage, as this can be measured and adjusted. So, if the generator voltage is expressed as:

(14) $$u_{G}=i_{G}\cdot X_{c}=\frac{i_{G}}{2\cdot \pi \cdot f\cdot C}$$

Substituting expression (14) into expression (13) gives:

$$P={\left(\frac{i_{G}}{2\cdot \pi \cdot f\cdot C}\right)}^{2}\cdot 2\cdot \pi \cdot f\cdot \varepsilon_{0}\cdot \varepsilon_{r}\cdot \frac{A}{d}\cdot \mathrm{tg}\delta \;$$

(15) $$P=\frac{i_{G}^{2}\cdot \mathrm{tg}\delta }{2\cdot \pi \cdot f\cdot C}$$

Furthermore, substituting expression (12) into expression (15), gives the expression for calculating the power level P via the anode current of the generator, i.e., the current of the upper electrode:

(16) $$P=\frac{i_{G}^{2}\cdot d\cdot \mathrm{tg}\delta }{2\cdot \pi \cdot f\cdot \varepsilon_{0}\cdot \varepsilon_{r}\cdot A}$$

To heat the weld from room temperature, or T_1_, to the melting temperature of the polymer material, T_2_, heat should be applied:

(17) $$Q_{H}=c\cdot m\cdot \left(T_{2}-T_{1}\right)$$

where:

Q_H_—heating the material from room temperature to melting temperature [J],
c—specific heat capacity of material [J/kg°C]
m—mass of the material to be welded under the upper electrode [kg]
T_2_—melting temperature of the polymer material [°C]
T_1_—room temperature/initial temperature of the material before welding [°C]

The mass of the material to be welded can be expressed as follows:

(18) m=ρ·V

where:

ρ—specific density of the material to be welded [kg/m^3^]
V—volume of the material [m^3^]

Since the volume of the weld seam the same as:

(19) V=A·d

where:

V—volume of the weld seam [m^3^]
A—area of the material [m^2^]
d—thickness of the material to be welded [m]

Substituting expression (19) into expression (18), the mass can be determined using the expression:

(20) m=ρ·A·d

In addition, substituting expression (20) into expression (17), the following is given:

(21) $$Q_{H}=c\cdot \rho \cdot A\cdot d\cdot \left(T_{2}-T_{1}\right)$$

The expression for latent heat or heat of melting must be defined for the polymer material to be welded:

(22) $$Q_{L}=m\cdot L$$

where:

Q_L_—latent melting heat of the welded polymer material [J]
m—mass of the material to be welded [kg]
L—latent heat of melting the material to be welded (specific melting heat) [J/kg]

Substituting expression (20) into expression (22) gives:

(23) $$Q_{L}=\rho \cdot A\cdot d\cdot L$$

The total heat Q_T_ required for welding polymer materials is the sum of specific heating and latent melting heat and is expressed as follows:

(24) $$Q_{T}=Q_{H}+Q_{L}$$

Equation (24) shows that the thermal cycle consists of two parts: the induction heating (QH), which raises the temperature of the weld from room temperature to melting temperature in the initial heating phase, and the heat of melting (QL) in the initial heating phase of the specimen, which leads to the transformation of the polymer material from a solid to a liquid phase [29].

Substituting expression (21) and expression (23) into expression (24) gives:

(25) $$Q_{T}=c\cdot \rho \cdot A\cdot d\cdot \left(T_{2}-T_{1}\right)+\rho \cdot A\cdot d\cdot L$$

That is:

(26) $$Q_{T}=\rho \cdot A\cdot d\cdot \left[c\cdot \left(T_{2}-T_{1}\right)+L\right]$$

Since Q_T_ = P∙t, where t is the time during which the HF energy acts on the weld and P is the power of this field, it follows that:

(27) $$t=\frac{Q_{T}}{P}$$

Substituting expression (26) and (13) into expression (27), gives an expression for the necessary time of influence of the HF energy on the weld, expressed as the work of the HF generator.

The total heat Q_T_ required for welding polymer materials can be calculated using expression (25), and the power of the influence of the HF energy on the weld using expression (14). Thus, by substituting expression (13) and (26) into (27), the expression for the required time of exposure of the HF energy to the weld seam is obtained, expressed by the voltage of the high frequency generator:

$$t=\frac{\rho \cdot A\cdot d\cdot \left[c\cdot \left(T_{2}-T_{1}\right)+L\right]}{u_{G}^{2}\cdot 2\cdot \pi \cdot f\cdot \varepsilon_{0}\cdot \varepsilon_{r}\cdot \frac{A}{d}\cdot \mathrm{tg}\delta \;}$$

(28) $$t=\frac{\rho \cdot d^{2}\cdot \left[c\cdot \left(T_{2}-T_{1}\right)+L\right]}{u_{G}^{2}\cdot 2\cdot \pi \cdot f\cdot \varepsilon_{0}\cdot \varepsilon_{r}\cdot \mathrm{tg}\delta \;}$$

Substituting expressions (26) and (16) into (27) in the same way, the expression for the required time of the influence of the HF power on the polymer joint is obtained, expressed by the anode current:

(29) $$t=\frac{\rho \cdot \left[c\cdot \left(T_{2}-T_{1}\right)+L\right]\cdot 2\cdot \pi \cdot f\cdot \varepsilon_{0}\cdot \varepsilon_{r}\cdot A}{{\;i}_{G}^{2}\cdot \mathrm{tg}\delta \;}$$

The expression (29) can be used to calculate the required exposure time of the HF generator when specifying welding current measured with an ammeter in the cathode circuit, as it is considered technically easier to achieve and relatively harmful (unlike the anode voltages, which can reach voltages up of to several kV). In the same way, the most important technological parameter, the time t, is determined, which is required for the influence of the HF energy on the weld seam, if all parameters relevant to the welding of polymer materials are known: specific density of the polymer material to be welded (ρ), thickness of the polymer material to be welded (d), specific heat to warm the material from room temperature to the melting temperature (Q_H_), heat of the polymer material to be welded (c), melting temperature of the polymer material to be welded (T_2_), room temperature/initial temperature of the material before welding (T_1_), latent melting heat of the welded polymer material to be welded (Q_L_), latent heat of melting the material to be welded or specific melting heat (L), total heat required for welding polymer materials (Q_T_), latent heat of melting the material to be welded (L), frequency of the HF generator (f), generator voltage (u_G_), anode current of the generator (i_G_), power of the generator (P), relative dielectric constant of the polymer material to be welded (ε_r_), area of the upper electrode (A), dielectric dissipation factor of the polymer material to be welded (tgδ), and welding time (s).

All the above parameters are known or can be measured before the technological process of welding the polymer materials and metals, and the required welding time can be determined in advance using expression (29). Then, by fine-tuning only two parameters, the welding time t and the anode current i_g_, the weld seam with the desired properties (visual appearance and strength of the weld seam) can be set.

Considering the previously listed key parameters of high frequency welding used in the mathematical expressions for the evaluation, it can be concluded that these are also most of the parameters on which the HF welding technology depends. In addition to the above parameters, we can also mention the area and shape of the electrode, the pressure force of the upper electrode, the maximum power of the HF generator, and the time required for cooling the specimen after welding.

## 2. Materials and Methods

In these studies, a polyurethane film Walopur 4201AU made by Bayer Epurex Films GmbH & Co. (Walsrode, Germany) [30], with a density of 1.15 g/cm^3^, a softening point of 140 to 150 °C and a very high elongation at break of 550 percent was used. In addition, the material is very UV-resistant, hydrolytically stable, has good thermal and ultrasonic joining properties, and good microbiological stability.

A measuring device and a measuring method have been developed to determine the breaking force using a tension meter and applying the T-peel test.

**Figure 2:**
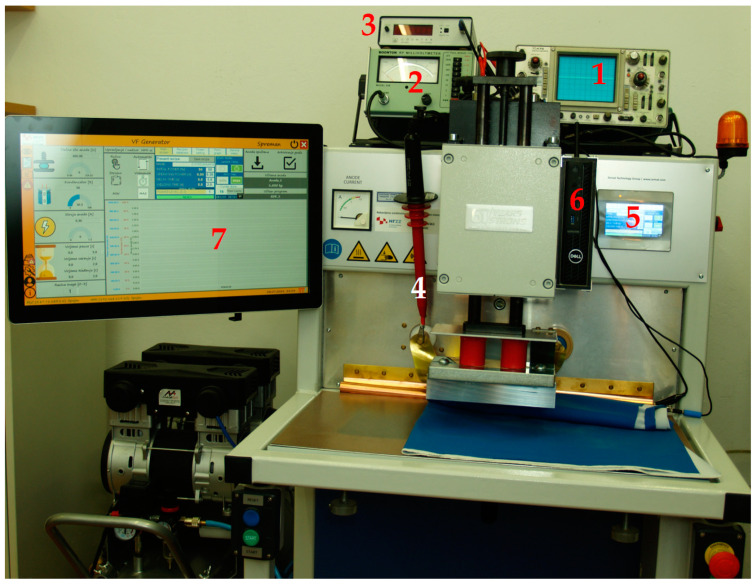
Machine for HF welding with additional measuring devices: 1—osciloscope; 2—voltmeter to measure effective values; 3—digital frequency counter; 4—high voltage probe; 5—small screen; 6—industrial PC; 7—touch screen monitor.

The investigation of the process parameters of HF welding was carried out on an HF welding machine ZD-N-4 Depta by Zemat Technology Group [31]. An HF machine was equipped with a linear pneumatic actuator to lower the upper electrode and regulate the pressure force, Figure 2, according to the idea of the first author of this article [32]. The figure shows the added instruments: an oscilloscope to measure the voltage waveforms at the upper electrode (1), an electronic voltmeter to measure effective values (2), and a digital frequency counter (3). All three of the above instruments are fed attenuated voltages through a high voltage probe (4).

On the right side below the oscilloscope there is a small screen (5) for displaying data with PLC control of the machine. The original machine with an integrated PLC circuit was upgraded with an industrial PC (6) for measuring and storing data and the application package for displaying the changes in process parameters as well as touch screen monitor (7) according to the idea of the first author of this article [32].

**Figure 3:**
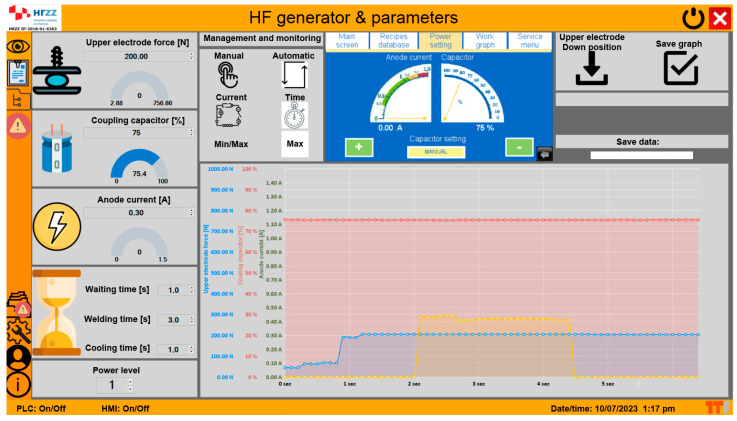
Screen layout of the monitor of the updated HF welding machine.

Figure 3 shows the screen of the updated HF welding machine. The screen shows the symbols of the most important control circuits and the currently stored values.

The force of the upper electrode can be adjusted from 0 to 750 N by changing the air pressure. The value of the coupling capacitor can be set in the range from 0 to 100%. This capacitor was designed as a coupling capacitor between the resonating circuit of the oscillator in the HF generator and the upper electrode. It has two functions: it protects the user of the machine from electric shock if the electrode is touched during positioning of the specimen, and it transfers the energy of the HF generator to the specimen when its values are changed. The value of the capacitance of the coupling capacitor (%) must be set via the servomotor and the PLC module before starting the welding work. The following time parameters can be set: the initial pause before the HF generator is activated (waiting time), the welding time when the HF generator is active, and the cooling time after welding. After welding, the heat is transferred from the weld seam to the electrode, which cools the specimen so that the chains in the macromolecular structure of the polymer reform. The value of the generator’s power level setting is also displayed (0—off; 1—1 kW power; 2—2 kW power, and 3—3 kW power).

A segment for selecting the type of machine control (manual, current, automatic, and time) appears on the screen and to the right of it a copy of the PLC screen with the parameters for the position of the upper electrode, the controls for archiving the parameter dependency graphs, and the control segments currently loaded from the memory for the welding recipes.

Most of the screen is used to display graphs of changes to the main welding process parameters in real time during the process. The pressure force of the upper electrode, the value of the capacitor, and the value of the anode current are recorded graphically. In addition to drawing the graphs, the measured values are stored in a matrix that can be read in numerical format and transferred to a programme for statistical analysis.

## 3. Results and Discussion

With the HF welding machine shown, numerous studies were carried out on functional variations in the process parameters, such as the influence of the percentage of the coupling capacitor, the anode current, and the welding time on the values of the breaking forces of welds produced using the HF welding technique with a constant upper electrode force of 200 N.

**Figure 4:**
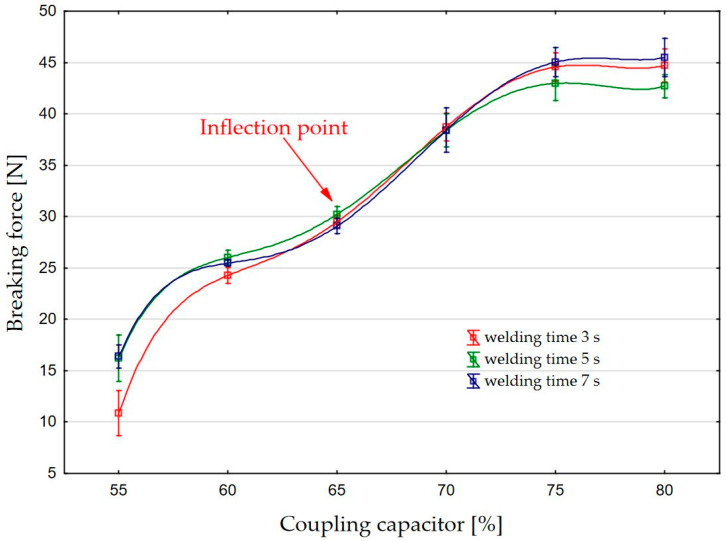
Dependence of the weld strength on the capacity of the coupling capacitor.

Figure 4 shows the graph of the dependence of the breaking forces on the capacity of the coupling capacitor of the HF welding machine for welding times of 3, 5, and 7 s. It can be seen that the values of the breaking forces for low values of the coupling capacitor at a value of 55% are relatively low (11 to 17 N). This can be attributed to an insufficient amount of the HF that could not be transferred to the upper electrode, i.e., to the specimen. For all values of the coupling capacitor from 0 to 50%, the supplied HF energy was too low to form a weld seam, so that the breaking force was 0 N.

As the capacitor value increases, the breaking force of the weld seam increases, i.e., the strength increases so that curve of interdependence of breaking forces and values of the coupling capacitor shows a continuous increase as more and more HF energy is transferred into the weld. At coupling capacitor capacity values higher than 80%, the energy injected into the weld is too high, causing the specimen to overheat and melt, decreasing its thickness due to the greater amount of extruded edges and creating an electric arc. The reduced thickness of the welded material cannot withstand the strong electric field, so an electric arc occurs. The material appears as a charred hole at the location of the arc and the machine’s safety system switches off the active working mechanisms, after which the first activation process must be carried out.

Figure 4 shows the inflection point.

The analysis of the inflection point identified during the experiment showed that the samples behave in two ways during the breaking force test: before and after the inflection point. When testing specimens where less energy was applied to the weld, it was observed that the weld is separated by peeling before the inflection point. These welds are usually low energy welds, where the extruded edges of the weld are less visible, and the weld has excellent visual properties. By adding more and more energy in a way that increases the value of the coupling capacitor or keeps it the same and extends the welding time, an increasingly rational appearance of the extruded edges is created behind the observed inflection point. Extrusion of the edges significantly worsens the aesthetic component of the joint, the joint becomes uneven and stiffer, and its elastic and flexible properties deteriorate [28,33]. Welds with pronounced extruded edges are not separated by peeling, but by tearing the material directly on one side of the extruded edge.

**Figure 5:**
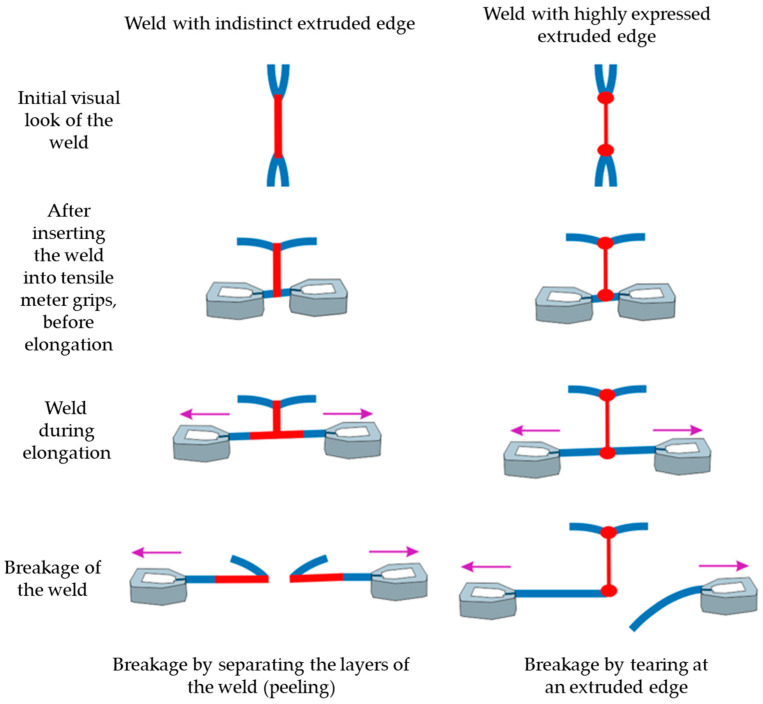
Phases of testing breaking forces of the HF weld.

Figure 5 shows a symbolic representation of the test phases and two characteristic types of tearing of HF welds, from the first visual appearance of the weld, after insertion into the tensile gauges, before elongation, during elongation, and at the break of the weld, both separate cases, peeling and tearing at the extruded edge.

In the first phase of the T-peel test, the specimen is not clamped with the tension gauge, so that the test force is not applied, and the specimen is not altered. In the second phase, the specimen is fixed in the clamps, the force is still not applied, and the specimen is not altered. In the third phase, the test force is applied. During a short period of time the force gradually increases, the specimen elongates, and after a certain time, the layers of the specimen separate. As the force gradually increases, a long elongation of the material is observed in some specimens before and while the layers of the weld separate by peeling. In the fourth phase, a fracture of the specimen is observed in the welded segment. There are two ways in which the weld seam separates from the specimen. One is peeling and the other is tearing of the material. Peeling means that each layer of the weld seam separates, and tearing means that the material breaks in the area where the extruded edge is located.

This unusual phenomenon of material separation of the HF weld seam (gradual peeling of the entire weld and tearing of the material in the area of the extruded edge) can be directly attributed to the amount of HF energy injected into the weld. As the original mathematical expressions for the evaluation by the authors of this article, the total energy Q_T_ injected into the weld is composed of expression (24), which is made up of the specific heat to heat the material from room temperature to the melting temperature Q_H_ and the latent heat of fusion of the material Q_L_. During the melting process of the weld seam, HF energy is first used to generate the specific heat and the temperature of the material, which rises rapidly until the melting temperature is reached. The energy is then converted into latent heat, as the temperature of the material does rise, but the material melts in its volume under the upper electrode.

The phenomenon described can be explained in the same way as the inflection point in the graph of the breaking force of the weld against the capacity of the coupling capacitor for values of the abscissa from 65% in Figure 4. The phenomenon of the inflection point for this abscissa value is very specific because the slope of the dependency curve of the welding force on the coupling capacity changes unexpectedly. In the range of values of the coupling capacitor from 55 to 65%, the dependence curve first rises rapidly and then slowly, and one might expect it to continue to rise slowly after the abscissa value of 65%, when it reaches a kind of saturation. However, upon visual observation of the HF welds breaking under various forces, the aforementioned characteristic separation of the weld was observed by gradual peeling and tearing in the location of the extruded edge. It was confirmed that this phenomenon of gradual separation of the HF welds by characteristic peeling occurs in the first part of the curve up to the inflection point. After this point, extruded edges are formed, an increase in breaking force is observed, and the quality of the welds decreases rapidly.

**Figure 6:**
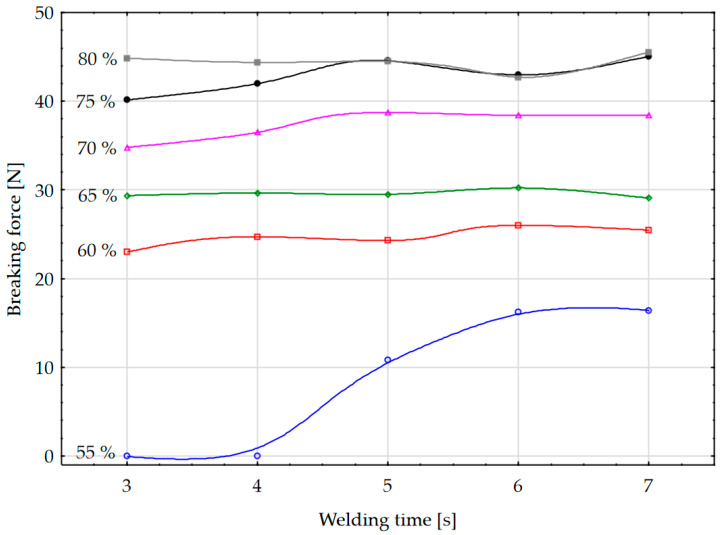
Dependence of the breaking forces of the HF welds on the connection time for different values of the capacity of the coupling capacitor.

From the graph of the dependence of the breaking forces of the HF welds on the welding time (F-t) for different values of the capacity of the coupling capacitor in Figure 6, the quantities of injected HF energy in the weld and the effects of the breaking forces of the weld, i.e., the strength of the weld, can be observed.

A particularly interesting graph is shown in Figure 6 for the lowest value of coupling capacitor capacity of 55% and welding times of 3 to 5 s. This is also the lowest amount of HF energy injected into the weld. It can be observed that a small amount of energy is injected into the weld during the welding time of the machine in the time range of 3 and 4 s and at the coupling capacitor capacity of 55%. In these short timeframes and with a small amount of HF energy of the generator, the energy was mainly used to heat the weld (by specific heat), but the material was not heated to the melting point; there was no melting of the material (latent heat phenomenon), so the material layers could not mix at the intermolecular level and the weld was not realised. Therefore, the breaking force is zero. After a welding time of the HF electromagnetic fields of 5 s, the amount of energy injected into the weld was sufficient so that the melting temperature was reached; the material layers started to melt and bond together, but only to a small extent, resulting in modest values of the breaking forces.

Increasing the values of the coupling capacitor can lead to sufficient energy being injected into the HF weld to form joints with higher breaking forces. At all coupling capacitor values of less than 50%, no two layers of the polymer material were welded together, and the breaking force was 0 N. Only after an increase of 55% could a breaking force value of more than 0 N be read. At values of 60% of the coupling capacitor, breaking forces of 25 N are achieved, at 65% around 30 N and with a particularly fine shape of the weld seam formed. With a continuous increase in the coupling capacitor value from 70 to 80%, a higher proportion of the HF energy is injected into the weld, which is unnecessarily used for unwanted melting of the material, resulting in poor properties and a poor technological process.

In accordance with the data obtained, it is considered that the amount of HF energy can be achieved by adjusting the value of the coupling capacitor, which means that a higher amount of energy is transferred to the upper electrode as the value of the coupling capacitor increases. Another possibility is to keep the value of the capacitor constant and increase the welding time. From a technical point of view, it is better to keep the welding time as short as possible, because with shorter times the load on the HF generator and the consumption of electrical energy are lower. From the technological point of view, this method is appropriate because it shortens the time of technological operation and increases production.

**Figure 7:**
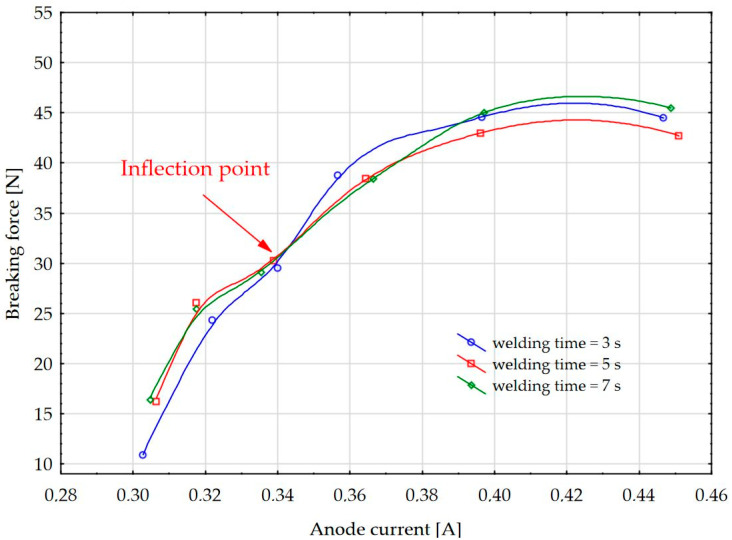
Dependence of breaking forces of the HF welds on the value of the anode current.

With these investigations it is possible to explain the dependence of the HF weld seam breaking forces on the values of the anode current (Figure 7). In the case shown in the graph, the anode currents for the time of the influence of the electromagnetic HF fields on the specimen are in the range of 0.30 to 0.46 A for a duration of 3, 5, and 7 s.

The graph also shows that the breaking forces of the weld seams increase as the anode current of the generator increases, up to a value of 0.42 A in the case shown. After that, the strength decreases because too much energy is injected into the weld seam, which can lead to dielectric arc in the weld. The inflection point is also clearly visible in this type of graph.

The graph of the dependence of the breaking forces on the anode current (F-i_g_) is more suitable for the scientific investigation of the process parameters of HF welding, but from a practical point of view it is easier to use the graph of the dependence of the breaking forces on the capacity of the coupling capacitor (F-C). This is because the value of the coupling capacitor can be changed during industrial operation of the process parameters by manual adjustment of the spindle and in computer-controlled machines with servomotors.

**Figure 8:**
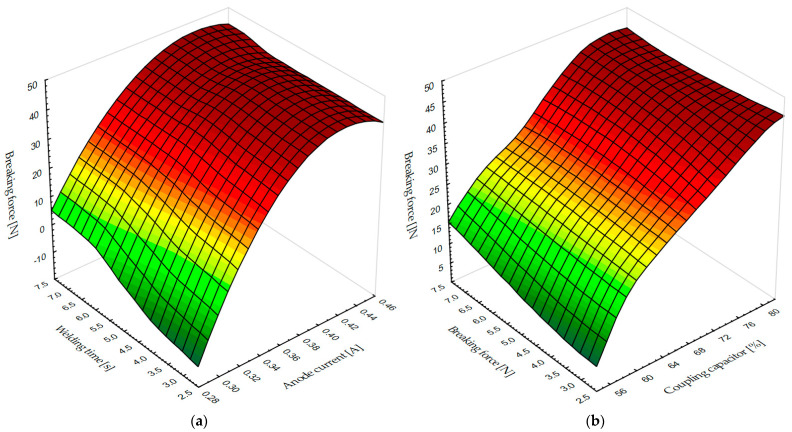
Graph of: (**a**) dependence of breaking forces of HF weld on welding time and anode current; (**b**) dependence of breaking forces of HF weld on welding time and coupling capacitor value.

On the basis of the investigation carried out, it is possible to draw not only a 2D graph but also 3D graphs of the dependence of the breaking forces of the HF weld on the welding time and the anode current, Figure 8a, or on the welding time and the coupling capacitor value, Figure 8b.

In both cases, the optimum path must be found so that the time of the HF electromagnetic fields is shorter and the values of the coupling capacitor or the anode current ensure an optimum breaking force and visual shape of the weld seam.

The extensive literature on the HF welding of polymeric materials shows that many authors consider three main parameters, namely the anode current, the welding time, and the upper electrode pressure. However, some authors have also worked with the welding frequency, the power, and the values of dielectric constants of the material or dielectric loss angle. In this research, the scope of parameters in the mathematical expressions for the evaluation is extended to 17 parameters that have proven to be crucial for the HF welding processes. Their mathematical relationships are shown, and formulas for calculating the parameters are given, as well as graph shapes showing the variation of many parameters that have been determined experimentally. From all this we can conclude that the field of investigation of process parameters of high frequency welding as one of the high-technology welding methods is very interesting and exciting, and that many more interesting studies can be expected in this field that will advance the development of clothing technology.

## 4. Conclusions

This article examines the dependence of the breaking forces in HF welding, i.e., the strength of the welds, on the values of the coupling capacitor, the anode current, and the welding time. Considering the dependence of the values of coupling capacitor, it was found that the breaking forces of the welds are low due to insufficient energy transfer to the specimen when using lower percentage values of the capacity of the coupling capacitor and at welding times. At higher capacity values of the capacitor, with which a higher amount of HF energy is injected into the weld, the breaking force of the weld, i.e., the strength of the weld, increases. A characteristic inflection point is observed, after which the properties of the weld seam deteriorate. When considering the dependence of the breaking forces of the weld seam, it was found that the breaking force increases when the anode current is increased. A further increase in the anode current leads to arcing at the weld seam because too much energy is injected into the weld seam. It is important to emphasise that from a practical point of view the use of the dependence of the weld strength on the capacitance of the coupling capacitor is easier to handle, since in production practice, the capacitance of the coupling capacitor spindle is adjusted more quickly manually or with computer-controlled machines. When testing the weld strength with the T-peel test, two types of HF weld fracture occur, namely, the gradual peeling of the entire weld and the tearing of the material at the extruded edge, which depends on the capacity of the coupling capacitor. It was found that the separation of the weld by peeling leads to a low proportion of extruded edges and the separation of the weld by tearing at the extruded edge leads to an increased proportion of extruded edges, characterised by higher strength. For this reason, process optimization of the weld formation is required. Based on the results shown in this paper it was found that the amount of HF energy can be regulated by adjusting the values of the coupling capacitor to achieve optimum weld strength, i.e., by increasing the energy transferred to the specimen or by fixing the capacitor value and increasing the welding time. It is necessary to determine the optimum balance so that the exposure time of the HF electromagnetic fields is as short as possible and the value of the coupling capacitor or the anode current ensures optimum breaking force and visual shape of the weld. From a technological point of view, it is better to shorten the welding time, as this shortens the duration of the technological process and increases productivity. With further research in the field of HF welding, it is possible to further develop the technology of fittings.

## Data Availability

Data are contained within the article.
